# Guided Bone Regeneration in the Edentulous Atrophic Maxilla Using Deproteinized Bovine Bone Mineral (DBBM) Combined with Platelet-Rich Fibrin (PRF)—A Prospective Study

**DOI:** 10.3390/jcm11030894

**Published:** 2022-02-08

**Authors:** João Manuel Mendez Caramês, Filipe Araújo Vieira, Gonçalo Bártolo Caramês, Ana Catarina Pinto, Helena Cristina Oliveira Francisco, Duarte Nuno da Silva Marques

**Affiliations:** 1 Instituto de Implantologia, Avenida Columbano Bordalo Pinheiro, n° 50, 1070-064 Lisbon, Portugal; fmaraujo.vieira@gmail.com (F.A.V.); caramesgoncalo@gmail.com (G.B.C.); anacatarina.pinto@institutoimplantologia.com (A.C.P.); helenafrancisco@campus.ul.pt (H.C.O.F.); duarte.marques@campus.ul.pt (D.N.d.S.M.); 2 Faculdade de Medicina Dentária, Universidade de Lisboa, 1600-277 Lisbon, Portugal; 3 LIBPhys-FCT UID/FIS/04559/2013, Faculty of Dental Medicine, University of Lisbon, 1600-277 Lisbon, Portugal

**Keywords:** platelet-rich fibrin, deproteinized bovine bone mineral, guided bone regeneration, horizontal augmentation, atrophic maxilla, natural scaffold

## Abstract

Background: Bone regeneration procedures represent a major challenge in oral surgery. This study aimed to evaluate a composite PRF/particulate xenograft in guided bone regeneration. Methods: Edentulous patients with horizontal ridge deficiencies in the anterior maxilla and candidates to an immediate-loading full-arch rehabilitation were included. Horizontal linear measurements indicating bone gain were assessed from computer beam computer tomography (CBCT) scans obtained at pre-surgery, post-surgery, and the 12-month follow-up. Mean bone values were presented as mean ± 95% CI. Non-parametric tests were used as appropriate, and the effect size was calculated with Cohen’s d repeated measures. Results: Eighteen patients were rehabilitated with 72 implants. The mean horizontal bone width was 4.47 [4.13–4.80] mm pre-surgically, 9.25 [8.76–9.75] mm post-surgically, and 7.71 [7.28–8.14] mm 12 months after. Conclusions: PRF associated with a xenograft seems to promote an effective horizontal bone gain. Randomized clinical trials are needed to confirm the benefits of this surgical approach.

## 1. Introduction

The atrophic maxilla represents a surgical and prosthodontic challenge for an implant-supported reconstruction [1,2]. Within the first 6 months following an anterior tooth extraction, the maxilla exhibits a primary volumetric change ranging from 29 to 63% horizontally and 11 to 22% vertically [3]. The presence of periodontal disease, trauma, other hard-tissue infections, or congenital malformations worsens this bone loss, which continuously progresses over the years following a centripetal pattern [4,5]. Cawood and Howell’s classification describes different stages of maxilla atrophy associated with the collapse of the circumoral musculature, triggering mouth narrowing, loss of lip support, and inversion of the lips [6]. This latter aspect is more significant in the anterior maxilla [7,8,9].

Despite the current perspective toward a minimally invasive graftless approach, it is recognized that most edentulous maxillae demand some type of bone augmentation procedure [10,11]. Implants in the graftless edentulous maxilla show high survival rates but are not exempt from side effects or prosthodontic complications [12]. Moreover, the level of bone atrophy has been reported to impact restorative and regenerative treatment schemes in a fixed full-arch rehabilitation [13]. It is clear that bone regeneration prior or simultaneously to implant placement in a fixed full-arch rehabilitation in the maxilla is essential not only for a prosthodontically driven concept but also to promote local bone defect and facial aesthetic corrections [6]. Many techniques, such as ridge splitting, bone block grafting, or guided bone regeneration (GBR) combined with several bone grafts (autografts, allografts, xenografts, and alloplasts) or different types of membranes or meshes have been described to horizontally reconstruct an alveolar ridge in the anterior region [14,15,16,17]. Despite the plethora of procedures, the most adequate option to obtain a stable horizontal bone dimension increase and a long-term implant and prosthesis survival rate is yet undetermined [15,18,19].

Based on high-evidence level publications, GBR seems more predictable, reproducible, and to result in fewer complications than other techniques [12,15,20]. GBR entails using a mechanical hindrance—usually a resorbable or a non-resorbable membrane—to separate the bony defect from epithelial and connective-tissue ingrowth, allowing creating a space for osteogenic cells to proliferate [21]. The need for optimizing horizontal bone gain and accelerating bone tissue formation has highlighted the importance of adding growth factors to GBR procedures [22].

A better comprehension of the cellular events that regulate bone tissue formation unveiled the rationale behind natural biological mediators. Accordingly, in order to enhance angiogenesis, stem cell migration, and osteogenic differentiation inherent to the regenerative procedure, several authors have suggested the potential of growth factors to bone grafts and tissue healing, mainly using autologous platelet concentrates (APCs) [22,23]. Since that, the efficacy of platelet concentrates in promoting wound healing and tissue regeneration has been the center of an enriched and participated academic debate. In 2005, Dohan et al. named a second generation of APCs as PRF, rich in platelets, leukocytes, and monocytes [24]. PRF is obtained according to a standardized protocol by venipuncture and 2700-rpm centrifugation for 12 min, without using anticoagulants [25,26]. It consists of a network of nanoscale fibers offering a natural scaffold for cell proliferation, migration, and differentiation [27]. Additionally, it slowly releases crucial growth factors during 10 to 14 days, such as PDGF, TGF-β1, IGF, and VEGF [27]. These factors play an important role in tissue healing [23]. According to a recent randomized clinical trial, PRF has proven to promote wound healing after dental extraction [28].

A liquid presentation of PRF, fibrinogen, can also be obtained by modifying centrifugation forces [27]. This flowable PRF may be regarded as an autologous binder, aggregating graft biomaterial, and chopped PRF membranes in a composite PRF/particulate xenograft as used in this prospective study. Clinical research has shown promising results from the association of PRF and particulate bone grafts in regenerative procedures [29]. However, according to a recent systematic review, limited studies investigate its application in intraoral bone grafting, especially in horizontal and vertical bone augmentation [30].

Based on the described background, this study aims to prospectively test deproteinized bovine bone mineral (DBBM) combined with PRF in GBR simultaneously to implant placement in edentulous patients with anterior maxillary atrophy who are candidates for immediate-loading full-arch rehabilitation.

## 2. Materials and Methods

### 2.1. Patient Selection

This study was a single-arm prospective clinical study with a pre-post design, carried out between December 2015 and January 2018. The study protocol was approved by the Local Ethical Committee with the ID: II 2015-06 and registered at ClinicalTrials.gov (U.S. National Library of Medicine, Bethesda, MD, USA) with the registration NCT03391258. All treatment phases were conducted per the Helsinki Declaration of 1975, as revised in 2008.

Eighteen patients were included in this study and signed the written consent forms before surgery. Patients were consecutively recruited according to the following inclusion criteria: (1) being over 18 years of age; (2) being ASA I or ASA II with mild systemic disease; (3) being edentulous or partially edentulous with failing teeth indicated for a fixed full-arch reconstruction; (4) having a horizontal bone defect in the anterior maxilla; (5) having an anterior ridge width allowing for lateral bone augmentation simultaneously to implant placement; (6) being compliant to participate in a clinical study. The exclusion criteria considered were: (1) ASA III or ASA IV; (2) oncologic history and/or chemo- and radiotherapy treatments in the previous two years; (3) having a horizontal bone defect caused by tumor resection; (4) pregnant women; (5) uncontrolled diabetes; (6) taking immunosuppressant medication or medication related to osteonecrosis of the jaw (MRONJ); (7) heavy smoking habits (over ten cigarettes); (8) hematologic diseases; (9) any type of psychiatric disorders; (10) participating in another clinical study.

The primary outcome measure was defined as the linear horizontal bone gain (mm) immediately after surgery and 12 months later, measured by CBCT post-surgically and at the 12-month follow-up. The secondary outcome measures were the rate of augmented bone stability at the 12-month follow-up, the incidence of post-operatory complications (soft tissue dehiscence, sensory disorder, wound infection, or graft exposure) recorded at 10 days and 1 month after surgery, and implant survival.

### 2.2. Outcomes Assessment

**Figure 1:**
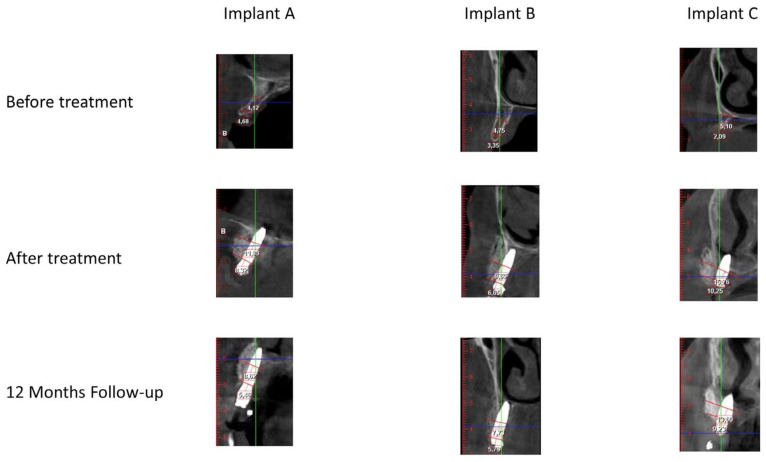
Example of measurements in three implant sites from different patients (**A**–**C**) evaluated through CBCT scans using the Planmeca Romexis^®^ Version 2.5.1.R. Images correspond to measurements in the different periods: before treatment, after treatment, and at the 12-month follow-up.

For each patient, CBCT (CBCT Planmeca ProMax Dimax 3 Digital Plan/Ceph) scans were obtained pre-surgery, immediately after the surgery (post-surgery), and at the 12-month follow-up. They were performed with a 0.20 voxel size, 80 kV, and 15 mA, within an exposure time of 12 s according to the manufacturer’s instructions. Cross-sectional images were reconstructed to a 0.6-mm thick slice with the artifact removal option applied. Two independent, calibrated operators (D.M.; A.P.) assessed each CBCT scan, and when in disagreement, mean values between their measurements were calculated. For the purpose of this study, only the pre-maxilla region, delimited by the anterior borders of the maxillary sinus, was analyzed, regardless of implant placement in the posterior maxillary areas. Four implants were analyzed per patient in the locations of the former lateral incisors and first premolars. In the sagittal cuts, each implant’s length was measured from the neck to the top. Site 1 for linear bone measurement was the neck of the implant, perpendicular to the long axis of the implant. Site 2 for linear bone measurements was the middle point of the total length of the implant. Both sites were measured from the most palatal point to the most buccal point (Figure 1A–C). The following variables were determined in sites 1 and 2, and mean values were calculated to assess the outcomes of this study.

Implant position in the pre-surgical CBCT scan was obtained from the post-surgical CBCT scan according to a previously described methodology [31]. On the axial view of the post-surgical CBCT, in the midline of the maxilla, the nearer anatomic reproducible landmark (nasal spine, incisive foramen, maxillary sinus anterior wall) was identified, and a straight line was drawn to the neck of the implant. The distance was obtained using the Planmeca Romexis^®^ software’s ruler and replicated in the pre-surgical CBCT scan. The pre-surgical width (mm) was measured from the cortical bone’s palatal point to the native bone’s buccal point, perpendicular to the long axis of the implant position. The post-surgical width (mm) was measured from the cortical bone’s palatal point to the regenerated bone’s buccal point, perpendicular to the long axis of the implant. The augmented horizontal bone width (mm) was calculated by the difference between pre- and post-surgical CBCT linear measurements. The same method was used to calculate the horizontal regenerated bone gain one year after surgery, using as reference the post-surgical and the 12-month linear measurements.

### 2.3. Clinical Procedures

In visit 1, inclusion and exclusion criteria were screened for each participant. After anamnesis and clinical examination, participants underwent preoperative prosthetic preparation (analysis of aesthetic features, such as facial profile, lip support, smile line, and residual ridge exposure). Subsequently, after the patient signed the informed consent, a pre-surgical CBCT scan (Planmeca Romexis^®^, Planmeca, Helsinki, Finland) was performed to evaluate maxillary resorption, which allowed planning for implant position and bone augmentation procedures.

**Figure 2:**
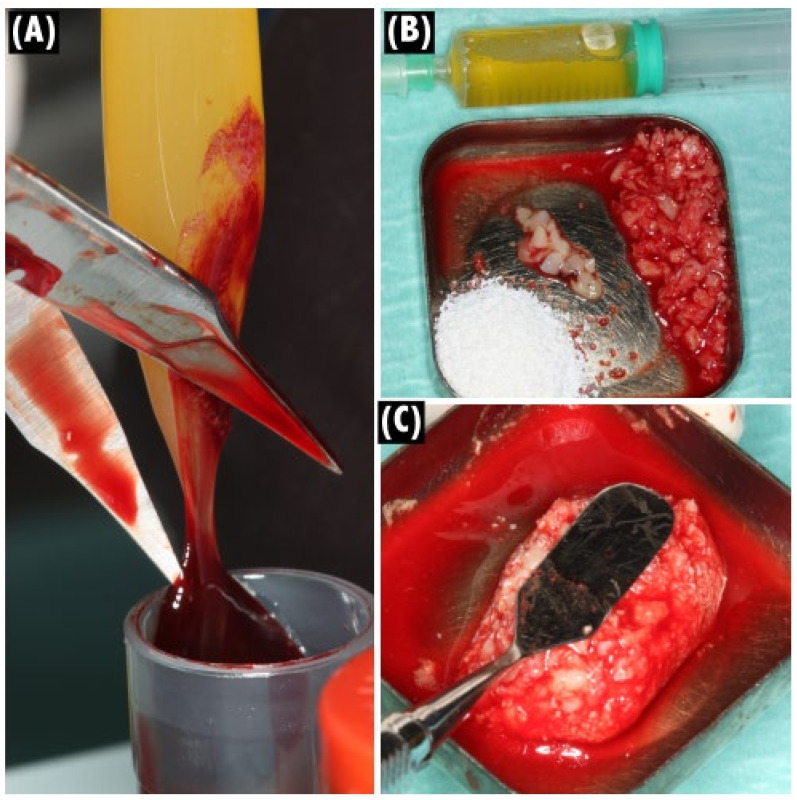
Clinical photographs of the protocol sequence to obtain the composite PRF/particulate xenograft: (**A**) Extraction of the PRF membranes from the red-topped tubes. The membranes were posteriorly pressed in a proper metal box; (**B**) Irrigation of autologous bone and xenograft with liquid fibrinogen obtained from the white-topped tubes; (**C**) Obtaining a composite PRF/particulate xenograft. Note the stiffness of this composite, which acquired a rectangular form.

**Figure 3:**
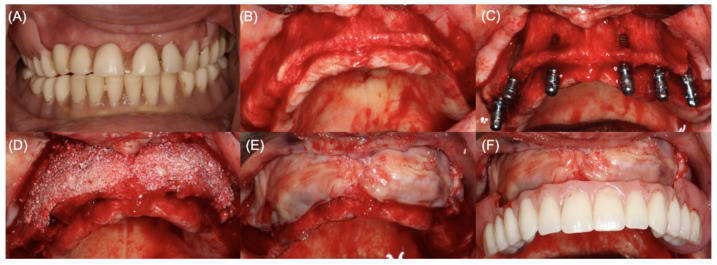
Clinical photographs of sequential surgical stages: (**A**) Atrophic maxilla of a partially edentulous patient who wore a removable prosthesis for more than 15 years; (**B**) Incision and full-thickness flap releasing. Note the thin buccal plate; (**C**) Implant placement. Note the buccal fenestration of the implants in the pre-maxilla; (**D**) Guided bone regeneration with a composite PRF/particulate xenograft to reconstruct the buccal aspect of the maxilla; (**E**) Overlay of the composite PRF/particulate xenograft with at least four PRF membranes, providing protection of the grafted area; (**F**) Full-arch implant-supported provisional restoration placed after surgery.

In visit 2, on the day of surgery, PRF membranes, a composite formed by particulate xenograft (Bio-Oss^®^ small particles, Geistlich AG, Wolhusen, Switzerland), and an average of eight PRF membranes and liquid fibrinogen were obtained according to a described protocol (Figure 2A–C) [26]. All surgical procedures were performed under strict sterile conditions, and each patient submitted to local anesthesia (articaine 4% with epinephrine 1:200,000, Inibsa, Sintra, Portugal), both buccally and palatally. A midcrestal incision was performed, followed by a mucoperiosteal flap release (total thickness) to expose the full extension of the buccal bone defect (Figure 3A,B). In partially edentulous patients, the remaining teeth were extracted in the least traumatic way to avoid tissue damage, and an osteotomy was performed to level the bone height and improve the prosthodontic design and features. Regardless of implant needs in the posterior region of the maxilla, four implants were placed in the former positions of the lateral incisors and first premolars (Straumann^®^ Bone Level Tapered Implants, Basel, Switzerland) with a minimum confirmed primary stability value of 45 N cm in each implant (Figure 3C). The composite PRF/particulate DBBM prepared was applied in the buccal plate of the pre-maxilla and shaped to restore bone volume. The grafted area was then covered with at least four layers of PRF membranes to protect it from exposure and optimize wound healing (Figure 3D,E). A tensionless primary closure was obtained through horizontal periosteal releasing, flap advancement, and simple sutures using a non-resorbable suture (6-0 polypropylene Perma Sharp^®^ Sutures, Hu-Friedy, Chicago, IL, USA).

**Figure 4:**
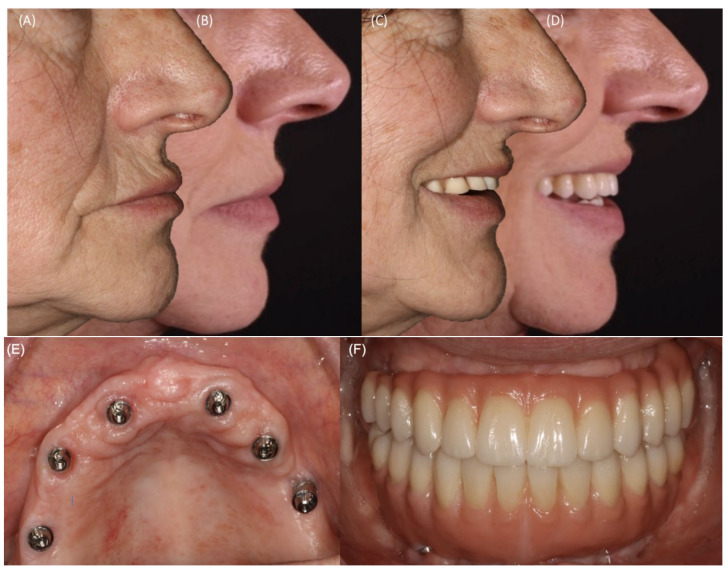
Clinical photographs reporting the relevance of the issue presented in this study. Evaluation of the aesthetic features related to lip support and prosthesis, as well as soft tissue healing and emergence profile at the 12-month follow-up appointment. (**A**) Lip support at rest before treatment; (**B**) Lip support at rest at the 12-month follow-up appointment; (**C**) Lip support at smiling position before treatment; (**D**) Lip support at smiling position at the 12-month follow-up appointment; (**E**) Emergency profile of the six implants placed in the maxilla. (**F**) Superior and inferior full-arch prosthesis in monolithic zirconia. Note that the maxillary prosthesis included a very reduced buccal flange due to the hard and soft regeneration obtained after surgery.

Immediate metal-reinforced fixed complete dentures were made, inserted, and adjusted on the same day of surgery to serve as immediate provisional restorations during healing and osseointegration (Figure 4F). Manufacture and insertion and adjustment procedures followed the principles described by Carames et al. [32]. Patients were medicated with amoxicillin + clavulanic acid 875/125 mg (starting the day before surgery with one tablet every 12 h, for 8 days), 600 mg of ibuprofen (every 12 h for 3 days), and 1 g of paracetamol (every 8 h, for the first three days) They were also instructed to rinse twice a day with chlorhexidine (Perio Aid 0.12%; Dentaid, Spain) mouth rinse and not brush the surgical area until suture removal at day 10.

Patients were submitted to mandatory post-surgical control appointments at 10 days, 1 month, and 6 months after surgery, and others if needed. At the 10-day and 1-month follow-up appointments, post-operatory complications, such as sensory disorder, infection, graft exposure, and dehiscence were assessed, and the number of events was determined. At the 1-year follow-up appointment, participants were also submitted to a clinical observation to monitor aesthetic features related to lip support as compared to baseline (Figure 4A–D), prostheses, soft tissue healing, and the emergence profile (Figure 4E,F).

### 2.4. Statistical Analysis

All the collected data were processed in SPSS software (version 24; IBM SPSS Statistics, Chicago, IL, USA) for statistical analysis. Results were expressed as a mean ± 95% confidence interval (CI) in millimeters for linear bone measurements or as a percentage for augmented bone. Normality of distribution was tested by Shapiro–Wilk’s normality test, and Levene’s test was used to assess the equality of variance. According to the variables to be assessed, the non-parametric Friedman’s, Mann–Whitney U, or Kruskal–Wallis tests were used to compare overall or different sites for regenerated bone linear changes (α = 0.05) using the patient as the statistical unit. The effect size was calculated with Cohen’s d repeated measures between pre- and post-surgical measurements and between post-surgical and 12-month follow-up measurements, and considered as small (<0.3), moderate (0.3–0.8), or large (≥0.8) effect. A sample size determination was performed to detect a large effect (≥0.8) in bone gain at the 12-month follow-up appointment, using G*Power 3.1 software (Heinrich-Heine-Universität, Düsseldorf, Germany). Considering a significance level of 5% and a power of 80%, a minimum of 12 participants was required. To offset a possible attrition bias, 50% was added to the total sample, resulting in 18 patients. An independent statistician performed the statistical analysis.

## 3. Results

The 18 participants included five men and 13 women, with ages ranging from 36 to 76 years (mean: 58 years). Seven were smokers (less than ten cigarettes per day). No dropouts were registered. This study assessed a total of 72 implants, of which one was lost during the follow-up period, equating to a survival rate of 98.61%.

**Figure 5:**
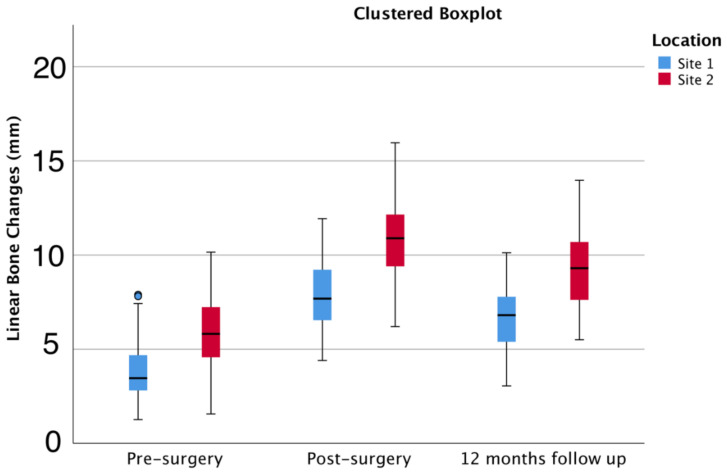
Linear bone changes (mm) (mean ± 95% CI) for different time points in 144 locations [*n* = 18 patients]. *p* < 0.05 between locations and time points.

Table 1 presents horizontal bone changes per site at designated time points: pre-surgery (before treatment), post-surgery (after treatment), and a 12-month follow-up. For both sites, the post-surgical and 12-month follow-up horizontal width was statistically higher (Friedman’s test, *p* < 0.05) than the pre-surgical values, with a d_repeated measures_ of 2.38 [2.07; 2.67] and 1.66 [1.39; 1.94], respectively. Site 2 also showed statistically higher values than site 1 at the post-surgical and 12-month follow-up CBCTs (Figure 5). The mean horizontal width at the 12-month follow-up was statistically different from the pre- and post-surgery values (Friedman’s test, *p* < 0.05), with a d_repeated measures_ of −1.12 [−1.38; −0.86] between the post-surgical and the 12-month follow-up horizontal widths. The obtained effect sizes were above the minimum detectable effect size of 0.098, with an alfa of 0.05 and a beta of 0.2. The overall mean augmented bone gain at post-op was 4.54 mm [4.09; 4.98] with statistically significant differences between 3.96 [3.51; 4.41] mm for site 1 and 4.96 [4.32; 5.60] mm for site 2 (Mann–Whitney U test, *p* < 0.05). No significant differences were detected when comparing augmented bone stability between sites (Mann–Whitney U test, *p* > 0.05) or implant locations in the aesthetic area (Kruskal–Wallis, *p* > 0.05).

None of the included patients presented complications during the follow-up visits (10 days and 12-month post-operation).

## 4. Discussion

The evaluation of facial changes, such as loss of lip support, has increasingly been considered when planning implant-supported rehabilitation of edentulous patients [33,34]. As previously reported, horizontal bone augmentation in immediate full-arch reconstructions is a frequent procedure that should also consider the patient’s preferences, expectations, and morbidity [13]. Although autogenous bone is regarded as the gold standard graft material due to its osteogenic, osteoinductive, and osteoconductive properties, it is also associated with increased morbidity, a limited amount of volume, and variable resorption rates [5]. Thus, from a patient-centered perspective, clinicians should avoid a second invasive harvesting surgery to collect autogenous bone. To overcome these limitations, research on tissue engineering and hard-tissue regeneration has escalated in the last two decades [35]. One of the strategies focused on growth factors, aiming to modulate cellular events involved in tissue healing and repair [35]. Platelet-rich fibrin is a second generation of APCs used in the management of severe medical conditions [23]. In dentistry, PRF was recognized as reducing post-operative pain, post-surgical bleeding in patients taking antiplatelet drugs, and promoting soft tissue epithelization [28,29]. Few studies have been identified regarding its use in horizontal or vertical bone regeneration [29,36,37,38]. To the authors’ best knowledge, this is the largest prospective clinical study conducting an immediate-loading protocol in the maxilla simultaneously with a GBR procedure using a mixture of particulate xenograft and PRF membranes embedded in liquid fibrinogen to form a composite graft.

The results of this prospective clinical study based on a sample size of 18 patients suggest that combining PRF membranes with particulate deproteinized bovine bone mineral can be effective and safe in treating horizontal bone defects in the anterior maxilla together with implant placement. The mean horizontal bone gain of 3.24 [2.85; 3.64] mm observed agrees with the main findings of systematic reviews of randomized clinical trials analyzing a GBR technique for lateral ridge augmentation [15,16,18,19,31,39]. The high heterogeneity between studies included in these SRs may explain slightly different outcomes between them [15,18,39]. Namely, Milinkovic et al. described a 3.31 mm horizontal bone gain when using GBR, whereas Elnayef et al. reported 2.59 ± 0.23 mm for the same approach [15,18]. Most of the included GBR studies used different types of bone grafts—either autologous, xenogenous, allogenous, or a mixture of both, with different types of membranes and in different edentulous regions. Moreover, several other factors may influence the mean horizontal bone gain outcome: regeneration technique, incision design, flap management, recipient site preparation, graft stability, tension-free primary closing, and defect morphology [5]. Ridge augmentation procedures are technique-sensitive and rely on the operator’s skillfulness and proficiency [5]. In this study, the previously referred aspects were consistently managed by a single experienced surgeon (J.M.M.C.), except defect morphology, intrinsic to the patient.

Regardless of the technique or biomaterials used, different levels of graft resorption are expected to occur [5,29]. Until now, few studies have provided information about bone graft stability at long-term follow-ups (≥12 months). However, it is relevant for long-term implant success and facial aesthetic corrections [18]. This study evaluated graft stability by comparing horizontal width at the 12-month follow-up with post-surgical width, and found a statistically different value (Friedman’s test, *p* < 0.05) with a d_repeated measures_ of −1.12 [−1.38; −0.86]. mm. A recent proof-of-concept study proposing the combination of PRF and DBBM in horizontal bone augmentation reported an average bone graft stability of 84.4% in a shorter follow-up period of 5 to 8 months [38]. Elanayef et al. also found a slight horizontal resorption of 1.13 ± 0.25 mm at the 6-month follow-up for GBR procedures. Estimating this value is extremely useful for predicting the surgical approach and properly overcorrecting the bone defect [40]. In the author’s opinion, this slow resorption rate is probably attributed to the presence of xenograft. The biocompatibility and osteoconductivity of DBBM are well documented in pre-clinical and clinical studies. However, a reduced osteoclastic activity toward this bone substitute is hypothesized [40]. In a clinical trial, a histomorphometric analysis showed that DBBM particles remained unchanged and integrated within the bone for 11 years [41].

GBR in maxilla bone augmentation procedures is usually associated with an implant survival rate (ISR) ranging from 96.1% to 100% [12]. Higher ISR values are obtained when GBR is performed simultaneously, and implants are placed in native bone [17,39]. It was the case in the present study, where a 98.61% ISR was obtained. Horizontal ridge augmentation with xenogenous bone graft is associated with a complication rate of 7.85% [19]. Membrane exposure is the most frequent complication in this type of intervention [19,40]. In this study, no complications were registered during the follow-up visits. However, no indications or conclusions can be withdrawn based on this finding in a single-arm clinical study. According to a systematic review, PRF membranes are associated with improved wound healing, soft tissue regeneration, and epithelization [42]. Thus, we might speculate that this advantage favored soft tissue healing after a horizontal bone augmentation procedure and subsequently avoided membrane and graft exposure. The standardization of the protocol to obtain PRF membranes and liquid fibrinogen was accomplished according to a published study and is an advantage of this research work [26].

The present study may entail some limitations. The primary outcome was assessed through linear measurements of CBCT scans. Several studies applied a similar methodology by measuring the horizontal bone gain in specific points of the alveolar ridge [31,36,43,44,45]. Although regarded as a reproducible method to evaluate bone gain obtained after GBR, dental implants may cause artifacts in CBCT images, reducing image quality and anatomic accuracy [46]. Although any blurred scan was subjected to artifact correction by the Planmeca ARA™ metal artifact removal algorithm, aiding operator’s measurements, this methodology is not exempt from limitations.

Since horizontal bone regeneration represents an added bone volume, the linear nature of the acquired measurements is a weak point in this study. Although the measurement landmarks are representative of the grafting area, a volumetric description variation would be preferable [38]. Based on this perspective and considering the development of high-speed and high-accuracy 3D intraoral scanners, their use in future studies may represent a less invasive option to evaluate bone and soft tissue volume gain.

In the GBR technique performed, PRF membranes were solely used as a barrier to cover the composite PRF/particulate DBBM placed in the anterior maxilla’s bone defect. Contrary to a similar study aiming at maxilla horizontal bone augmentation, no resorbable collagen membrane or fixation pins were used for membrane stabilization under PRF membranes [38]. When using particulate bone substitutes in large defects like those regenerated in the present study, membrane stabilization for graft immobilization represents a challenge and key factor for GBR success [17,40]. This objective was mainly achieved in this study by adding liquid fibrinogen binding bone graft particles in the composite PRF/particulate DBBM graft. In addition, the PRF membrane cross-linking structure provided an elastic mechanical behavior suitable for membrane handling and graft covering [27]. Non-resorbable membranes fixated with pin systems show less tendency to collapse and maintain the horizontal contour of the augmented ridge [17]. However, this type of approach presents more complications, especially membrane exposure, and requires additional surgery to remove the membrane and the pins when patients subjected to immediate full-arch rehabilitation usually do not expect additional surgery procedures. Immediate-loading simultaneously to bone regeneration has been poorly described in the literature [47]. Although immediate loading in maxilla full-arch rehabilitation can be a predictable protocol when key factors are achieved, such as adequate primary implant stability, a favorable drilling technique, implant design, length, and implant microstructure [48,49]. In addition, the importance of osteotomy techniques should be recognized when a minimum insertion torque of 45 N·cm is obtained in a more medullary bone [50]. A clear influence of a bone compactor preparation to implant primary stability was suggested by Attanasio et al. in this type of bone [50]. In this study, the operator (J.M.M.C.) followed brand instructions and accomplished an under-preparation scheme, promoting a more frictional implant insertion. Other aspects, such as cross-arch stabilization, prosthetic material, and prosthetic rehabilitation design aided to unload the regenerated area—a crucial aspect of the bone regeneration process.

Because this study is a single-arm prospective clinical study without any comparison group, the conclusions regarding the use of PRF in horizontal bone augmentation are limited. An RCT study design would have the potential to better clarify the discussed biological benefits. A major aspect of the biological plausibility proposed for PRF in bone regeneration should also be questioned. Fibrin matrix degradation and growth factor release occur in the first 10 to 14 days, whereas bone tissue formation occurs in 3 to 4 months [27]. It is easily perceived that this natural scaffold lacks a spatial-temporal delivery of growth factors concomitant to bone formation events. Since it derives from blood, its specificity, and minimum concentration of growth factors to aid osteogenesis’s initial phases are still unknown. Therefore, future research should aim to clarify this question and optimize scaffolds prepared in order to assist all events of bone tissue formation, providing a combined release of growth factors and extracellular matrix components.

## 5. Conclusions

Within the limitations of this clinical study, the use of composite PRF/particulate xenograft in GBR for horizontal bone augmentation with simultaneous implant placement seems safe and provides a horizontal bone gain and graft stability within previously described values for GBR procedures. This result might be particularly relevant since no resorbable collagen membranes were used to cover an immobilized bone graft obtained by adding liquid fibrinogen. No type of complication has been registered. Further studies are needed to validate or exclude the potential biological benefits of PRF in GBR procedures for maxilla horizontal bone augmentation.

## Figures and Tables

**Table 1:** Linear bone changes (in mm) for each location and overall at different evaluation times.

		Pre-Surgery	Post-Surgery	12-Month Follow-Up	Bone Gain (12 Months—Pre-Surgery)
Site 1	Mean	3.52	7.79	6.53	3.01
	95% CI	3.19–3.85	7.30–8.28	6.09–6.96	2.51–3.50
	Range	6.16	7.53	6.6	8.17
Site 2	Mean	5.5	10.84	8.99	3.5
	95% CI	5.04–5.95	10.19–11.48	8.42–9.57	2.87–4.13
	Range	8.59	9.76	8.47	8.46
Total	Mean	4.47	9.25	7.71	3.24
	95% CI	4.13–4.80	8.76–9.75	7.28–8.14	2.85–3.64
	Range	8.89	11.56	10.92	8.5

## Data Availability

Data cannot be shared due to data protection obligations.

## Abbreviations

PRF	Platelet-rich fibrin
CBCT	Cone beam computed tomography
GBR	Guided bone regeneration
APCs	Autologous platelet concentrates (APCs)
PDGF	Platelet derived growth factor
TGF-β1	Transforming growth factor β1
IGF	Insulin-like growth factor
VEGF	Vascular endothelial growth factor
DBBM	Deproteinized bovine bone mineral
ASA	American Society of Anesthesiology (physical status classification score I–VI)
MRONJ	Medication related to osteonecrosis of the jaw
SRs	Systematic reviews
ISR	Implant survival rate
RCT	Randomized clinical trial
